# Memory performance following napping in habitual and non-habitual nappers

**DOI:** 10.1093/sleep/zsaa277

**Published:** 2020-12-12

**Authors:** Ruth L F Leong, Nicole Yu, Ju Lynn Ong, Alyssa S C Ng, S Azrin Jamaluddin, James N Cousins, Nicholas I Y N Chee, Michael W L Chee

**Affiliations:** Centre for Sleep and Cognition, Yong Loo Lin School of Medicine, National University of Singapore, Singapore; Centre for Sleep and Cognition, Yong Loo Lin School of Medicine, National University of Singapore, Singapore; Centre for Sleep and Cognition, Yong Loo Lin School of Medicine, National University of Singapore, Singapore; Centre for Sleep and Cognition, Yong Loo Lin School of Medicine, National University of Singapore, Singapore; Centre for Sleep and Cognition, Yong Loo Lin School of Medicine, National University of Singapore, Singapore; Donders Institute for Brain, Cognition & Behaviour, Radboud University Medical Centre, EN, Nijmegen, The Netherlands; Centre for Sleep and Cognition, Yong Loo Lin School of Medicine, National University of Singapore, Singapore; Centre for Sleep and Cognition, Yong Loo Lin School of Medicine, National University of Singapore, Singapore

**Keywords:** naps, habitual, adolescents, memory, learning

## Abstract

**Study Objectives:**

Afternoon naps benefit memory but this may depend on whether one is a habitual napper (HN; ≥1 nap/week) or non-habitual napper (NN). Here, we investigated whether a nap would benefit HN and NN differently, as well as whether HN would be more *adversely* affected by nap restriction compared to NN.

**Methods:**

Forty-six participants in the nap condition (HN-nap: *n* = 25, NN-nap: *n* = 21) took a 90-min nap (14:00–15:30 pm) on experimental days while 46 participants in the Wake condition (HN-wake: *n* = 24, NN-wake: *n* = 22) remained awake in the afternoon. Memory tasks were administered after the nap to assess short-term topographical memory and long-term memory in the form of picture encoding and factual knowledge learning respectively.

**Results:**

An afternoon nap boosted picture encoding and factual knowledge learning irrespective of whether one habitually napped (main effects of condition (nap/wake): *p*s < 0.037). However, we found a significant interaction for the hippocampal-dependent topographical memory task (*p* = 0.039) wherein a nap, relative to wake, benefitted habitual nappers (HN-nap vs HN-wake: *p* = 0.003) compared to non-habitual nappers (NN-nap vs. NN-wake: *p* = 0.918). Notably for this task, habitual nappers’ performance significantly declined if they were not allowed to nap (HN-wake vs NN-wake: *p* = 0.037).

**Conclusions:**

Contrary to concerns that napping may be disadvantageous for non-habitual nappers, we found that an afternoon nap was beneficial for long-term memory tasks even if one did not habitually nap. Naps were especially beneficial for habitual nappers performing a short-term topographical memory task, as it restored the decline that would otherwise have been incurred without a nap.

**Clinical Trial Information:**

NCT04044885.

**Statement of Significance**

About half the general population habitually takes naps. We investigated whether a midafternoon nap differentially benefits habitual and non-habitual nappers. We also examined whether habitual nappers would be more adversely affected by *nap restriction*. Compared to staying awake, an afternoon nap was beneficial for long-term memory even if one was not a habitual napper. However, naps were especially beneficial for habitual nappers performing a short-term topographical memory task, as it restored the decline that would otherwise have been incurred without a nap. Our findings support the implementation of naps in educational settings in order to boost and protect learning and memory in school-going adolescents.

## Introduction

Afternoon naps have been shown to benefit memory in both adults [1–5] and adolescents [6, 7]. In the laboratory, napping after learning enhances memory consolidation [8–11] while napping before learning improves the ability to encode material [11, 12]. Despite these well-documented benefits, not everyone naps on a regular basis. However, compared to working adults who may have limited opportunity to nap in the day [13], 40%–60% of adolescents report napping at least once a week [14] and may be considered “habitual nappers” [15]. In addition, more adolescents nap on weekdays compared to weekends [14, 16]. This coincides with the finding that higher frequency of daytime napping is associated with shorter nocturnal sleep [17, 18], suggesting that teens who nap regularly may do so to make up for inadequate nocturnal sleep [19–21].

At present, most studies investigating naps do not control for nap habit, or may select only habitual nappers [22, 23] or non-habitual nappers [24–27] for practical reasons. Yet, studies suggest that those who are more accustomed to napping may experience naps differently. For example, habitual nappers may obtain more stage 1 sleep during a nap [28, 29] and report feeling more refreshed upon waking [30, 31], while those who rarely nap have high amounts of N3 when they do nap [29]. However, few studies have experimentally examined whether nap habit influences the learning and memory outcomes of a nap.

In young adults, a 20 min nap comprising mainly stage 1 and 2 sleep was found to be detrimental to non-habitual nappers learning a “cup and ball” motor memory task [32]. However, this short nap did not improve motor learning for habitual nappers either. The only other study to examine this question in young adults used a 90-min nap opportunity, allowing for a full cycle of sleep. They found that compared to staying awake, a 90-min nap improved perceptual learning to a greater extent in habitual nappers compared to non-habitual nappers [33]. However, as the performance of the habitual and non-habitual nappers who remained awake was not compared, it is unclear whether habitual nappers were also more adversely affected by *nap restriction* compared to non-habitual nappers. To this point, preschoolers aged 3–6 years who napped regularly experienced greater wake interference when kept awake in the afternoon compared to those who were not in the habit of napping [34]. However, it remains an open question whether this can be extended to adolescents who are at a different stage of brain development and who may have a lower propensity to nap [35, 36].

So far, these existing studies have only assessed memory consolidation. Given that boosting encoding may be especially advantageous for long-term learning outcomes in adolescents [37], we examined nap effects on encoding in a group of older adolescents varying in nap habit (15–19 years). Three different memory tasks were used: a short-term topographical memory task, and two long-term memory tasks probing picture encoding and factual knowledge learning. Firstly, we compared the benefits of a nap relative to wake in habitual and non-habitual nappers across these short-term and long-term memory tasks. While we predicted that naps would have an overall benefit on memory encoding, we expected that habitual nappers would benefit more from a 90-min mid-day nap following previous findings [33]. Polysomnographic measures were also obtained during the nap period to examine if differences in sleep architecture could explain any differences in group performance. Secondly, we compared the performance of habitual nappers who were deprived of their midday nap against non-habitual nappers who also remained awake. Based on earlier findings in a younger group [34], we anticipated that habitual nappers would experience more detrimental effects of nap restriction.

## Methods

### Participants

114 participants (mean age ± *SD*: 16.49 ± 0.97 years; 59 females) recruited from the Need for Sleep 4 [38] (NFS4) and Need for Sleep 5 studies [39] (NFS5). Participants had no personal history of sleep disorders, neurological, psychological, or other chronic illness, consumed <5 caffeinated beverages a day, and were not habitual short sleepers (i.e. individuals with <6 h of actigraphically assessed average time in bed [TIB] and no evidence of sleep extension for >1 h on weekends). All participants gave informed consent to participate, in compliance with a protocol approved by the National University of Singapore Institutional Review Board.

As part of the screening procedures to evaluate habitual sleep patterns, participants filled in a sleep diary for at least 1 week, in which they were also required to indicate if they took any naps. We classified habitual nappers as those who reported napping at least once a week, and non-habitual nappers as those napping less than once a week [32, 33]. We accounted for the variation in the total number of days each participant filled out their diary by using the proportion of naps taken to the total number of days the diaries were filled out. As such, habitual nappers were defined as those who had a nap frequency of ≥14.3% (corresponding to napping at least once a week), and non-habitual nappers of <14.3%. Habitual nappers reported napping an average of twice a week (range: 1–6 naps/week) for an average of 84.3 min. Although there was a wide range of nap durations reported (5–383 min), participants most often reported taking naps of 31–60 min. Naps most commonly occurred between 12:00 and 17:59 pm in the afternoon.

Participants were randomly assigned to a nap or wake condition. After excluding participants with incorrectly filled or incomplete sleep diaries as well as one participant who had missing data across the tasks, our final sample consisted of 92 participants (mean age ± *SD*: 16.49 ± 0.97; 48 females), with 46 participants in the nap condition (habitual nappers [HN-nap]: *n* = 25, non-habitual nappers [NN-nap]: *n* = 21) and 46 participants in the wake condition (habitual nappers [HN-wake]: *n* = 24, non-habitual nappers [NN-wake]: *n* = 22).

## Study Design

The memory tasks investigated were conducted during the NFS4 and NFS5 15-day protocols [38, 39], which aimed to track adolescents’ cognitive performance across two sets of nap and no nap schedules. Both studies’ protocols examined whether incorporating a midday nap into one’s sleep schedule would benefit cognitive performance and memory. Because both NFS4 and NFS5 involved a 90-min nap opportunity at the same time of day and nap sleep architecture was highly similar across studies (Supplementary Table 1), data from the same memory tasks performed on the same manipulation days across both studies were combined.

Participants in the Nap conditions were given a 90-min nap opportunity (14:00–15:30 pm) on manipulation days, while those in the wake conditions slept the equivalent total TIB entirely at night. The first cycle consisted of five days of the nap manipulation, followed by two non-manipulation (9 h nocturnal TIB) rest days. The second cycle included 3 days of nap manipulation and ended with two rest days for both studies. The memory tasks in the present investigation were administered on the third and fifth days in the first manipulation cycle, and across the three days in the second cycle (details below).

## Procedure

Prior to the experimental phase of each study, participants’ sleep patterns were assessed for at least a week with wrist actigraphy. During the screening session, other measures probing participant characteristics were obtained via a battery of self-reported questionnaires. The Raven’s Progressive Matrices (RAVENS) was used to assess non-verbal intelligence [40], the Epworth Sleepiness Scale [41] (ESS) was used to examine levels of daytime sleepiness, the Pittsburgh Sleep Quality Index [42] (PSQI) measured self-reported sleep quality, and the Morningness–Eveningness Questionnaire [43] was used to evaluate chronotype. The Beck Depression Inventory [44] (BDI) and the Beck Anxiety Inventory [45] (BAI) were also used to screen for levels of depression and anxiety respectively.

For a week prior to the experimental protocol, all participants adhered to a 9 h sleep schedule (23:00 pm–8:00 am) to minimize the effects of prior sleep restriction. Compliance was verified with actigraphy. The 15-day experimental protocols took place under close monitoring in a boarding school during school vacation time. Participants had twin-share bedrooms and all cognitive testing occurred in specified classrooms via individual laptops. They were seated approximately 1-m apart across six rows and were instructed not to look at other screens during the tasks.

**Figure 1.**
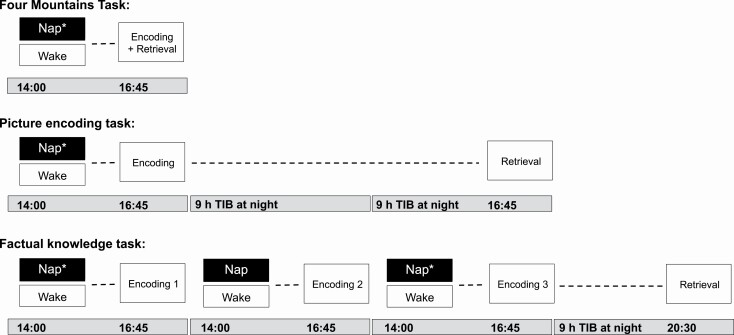
Memory task protocols. All encoding sessions were performed at 16:45 pm, approximately 75 min after the nap period (14:00–15:30 pm). In the Four Mountains Task (4MT), encoding was immediately followed by retrieval. For the picture encoding task, the retrieval session was performed after two nights of 9 h nocturnal time in bed (TIB). Encoding sessions for the factual knowledge task took place across 3 days, with encoding of material occurring after the nap period. The retrieval session took place in the evening (20:30 pm) of the next rest day. Asterisks indicate the naps in which sleep was assessed with polysomnography.

The memory tasks were administered on the same experimental days and at the same clock times across both NFS4 and NFS5 protocols (Figure 1). In order to examine the effects of a nap on encoding and memory, all learning sessions were performed at 16:45 pm, approximately 75 min after the nap period (14:00–15:30 pm). Three memory tasks were examined: (1) the Four Mountains task (4MT), a measure of short-term topographical memory, which took place on the third day of the first manipulation cycle, (2) the picture encoding task, measuring long-term episodic memory, which occurred on the last day of the first manipulation period, with retrieval performed after two days of rest, and (3) the factual knowledge task, a measure of long-term memory, which took place across the subsequent 3-day manipulation period. The factual knowledge retrieval test took place in the evening of the following rest day.

In addition, just prior to each encoding and retrieval session, participants completed a cognitive test battery which included the *n*-back task and psychomotor vigilance task (PVT) as measures of working memory and sustained attention. Details of the *n*-back task and PVT are provided in the supplementary materials.

## Polysomnography

Portable SOMNOtouch polysomnography (PSG) devices (SOMNOmedics, GmbH, Germany) were used to record sleep during the nap. EEG was recorded from two channels (C3 and C4 according to the 10–20 system). Contralateral mastoids were used as references. The common ground and reference electrode were placed at Fpz and Cz. Left and right electromyogram and electrooculogram were attached. Impedance <10 kΩ was verified at each electrode and the sampling rate was 256 Hz.

PSG data were scored according to criteria set by The AASM Manual for the Scoring of Sleep and Associated Events [46] using the Z3 score automated EEG scoring system [47] and verified by a trained researcher. The following nap macrostructure parameters were evaluated: total sleep time (TST), duration spent in individual sleep stages (N1, N2, N3, and REM), sleep efficiency, wake after sleep onset (WASO), as well as N2 sleep latency (time from lights off to N2 sleep onset) and sleep stage upon awakening.

Slow-wave activity (SWA; 0.6–4 Hz) was also computed following prior work [48] on non-overlapping, artifact-free 5 s epochs using custom routines in Matlab R2016b (The MathWorks, Inc. Natick, MA). To do this, power spectral density estimates for each epoch were computed using Welch’s modified periodogram method [49] (Hamming window; 0.2 Hz bin resolution) and integrated from 0.6 to 4 Hz using the trapezoidal rule for an integral approximation to obtain to SWA measures per epoch. Mean SWA per nap record was then computed by averaging across all NREM epochs. Only records containing <10% of artifacts were included in subsequent analysis, and the C3-A2 electrode was selected for consistency with prior work.

To further explore significant findings pertaining to nap effects, we performed automatic sleep spindle detection analysis on the nap period preceding the relevant task using the Wonambi Python package, v5.24 (https://wonambi-python.github.io) with an algorithm developed by Molle et al. [50]. In brief, the EEG was filtered between 12 and 15 Hz, and then the root-mean-square (RMS) of the signal was computed at every sample point using a moving window size of 200 ms. Spindle events were detected as a continuous rise in the smoothed RMS signal above 1.5 × standard deviations of the smoothed RMS signal, lasting between 0.5 and 3 s. Spindle count and density (per min) were computed for NREM epochs from both C3-A2 and C4-A1 electrodes, but as results did not significantly differ between electrodes, we only present findings here for C3-A2.

## Four Mountains Task

The 4MT measured short-term topographical memory [51], the processing of which has been shown to be hippocampal-dependent [51, 52]. This task was programmed in E-Prime 2.0 (Psychology Software Tools, Inc, Sharpsburg, PA). This task took place 75 min after the nap manipulation period at 16:45. Each trial started with a 10-s presentation of a sample landscape with a unique topography, depicting four mountains that varied in size, shape, and relative distance from each other. A 7-s blank screen followed this, after which a four-alternative choice of landscapes arranged in a 2 × 2 grid was presented for 20 s. Participants were required to draw upon their topographical memory to correctly select the previously shown landscape, which now appeared from a different viewing orientation (i.e. the same landscape has previously shown but taken from a different virtual camera position). The three foil images had the same viewpoint and non-topographical features (color and texture of surfaces, atmospheric conditions, cloud cover, and sunlight direction) as the target image, but foil landscapes comprised mountains of different sizes, shapes, and relative locations. The on-screen position of target and foil images was randomized for each trial.

Each participant chose the landscape response alternatives with different keys (“Q,” “W,” “A,” or “S”). Trials were presented in a single randomized block lasting approximately 16 min. Task performance, as assessed by accuracy, was indexed by the proportion of correct responses.

## Picture Encoding Task

Stimuli used in this task, selected from Takashima et al. [53] included 240 images of a variety of building types and landscapes. Half of the images contained buildings, while the other half contained no buildings. These images were split into three sets of 80 (40 buildings, 40 no buildings). Two of the sets were presented during encoding and retrieval (160 old images), while the remaining third set was presented only at retrieval (80 new distractor images).

The encoding session took place after the nap manipulation period at 16:45 pm in a single block of approximately 15 min. Participants were instructed to look at each image and determine whether there was a building in it or not by responding with a keyboard press. They were not told that their memory on these would be tested at a later date. The retrieval session took place after two rest nights of 9 h TIB at 16:45 pm. This tested participants’ recognition of the 160 old images from the encoding session, randomly intermixed with 80 new images. Participants indicated their responses using specific keys. When probed, none of the participants reported anticipating the retrieval test.

Any images during encoding that were incorrectly judged as containing buildings or not were excluded from retrieval analysis, as these trials may not have been properly attended to. The non-parametric signal detection measure A′ was calculated using hits and false alarm rates in order to account for participants’ response bias toward old or new responses with 0.5 indicating chance level performance. The full task paradigm has been previously described [54].

## Factual Knowledge Task

The factual knowledge task was used as a measure of long-term memory (see Cousins et al. [55] for full details). A pre-test was performed prior to learning to assess for pre-existing knowledge of the species. All trials were presented in random order and all tests were self-paced.

Encoding of the substantial amount of material took place across three sessions over 3 days that occurred after the nap period at 16:45 pm. Participants were informed that they would be tested on the facts they were about to learn. In the afternoon learning session following the nap period, participants learned facts about six species of amphibian that were repeated each day (selected from three toads, three newts, three frogs, and three salamanders). The species learned were counterbalanced across participants. The one-hour learning sessions were split into 30-min blocks separated by a 2-min break.

Retrieval took place after a night of 9 h TIB in the next evening at 20:30 pm and consisted of two-alternative choice questions followed by a confidence rating (“certain,” “somewhat certain,” “guess”). These were presented randomly within six blocks that were separated by 30 s breaks. In line with previous studies showing that certain scores are the least prone to noise introduced by participants’ guessing [55], we examined only certain responses and corrected for response bias (correct – incorrect).

### Statistical analyses

All analyses were performed with SPSS 25.0 (IBM, Chicago, IL). As the present study investigated the factors of nap habit group, that is, habitual nappers (HN) and non-habitual nappers (NN), as well as the experimental conditions of nap and wake, we refer to the combinations of groups and conditions as HN-nap, HN-wake, NN-nap, and NN-wake.

We first used independent samples *t*-tests and chi-squared tests to determine if HN and NN statistically differed in screening variables and actigraphically assessed sleep patterns. Next, we examined if habitual and non-habitual nappers assigned to the nap groups (HN-nap, NN-nap) differed in their sleep architecture during each of the four polysomnographically assessed naps that preceded the learning sessions (see Figure 1). Fisher’s exact test was used to examine if habitual and non-habitual nappers differed in their likelihood of awakening from the naps at N1, N2, N3, or REM sleep stages.

In order to determine if the effects of naps and wake varied depending on whether one was a habitual napper, we performed two-way ANOVAs with the between-subject factors of group (habitual nappers [HN], non-habitual nappers [NN]) and condition (nap, wake) for performance on the 4MT, picture encoding task, and the factual knowledge task. Group contrasts were tested with independent samples *t*-tests. In addition, to investigate if group differences in post-nap vigilance and working memory could account for our findings, we used similar two-way ANOVA models for PVT lapses (response times > 500 ms), PVT median response times as well as n-back performance (results in Supplementary Analysis). Lastly, we followed significant interactions with Spearman’s rho correlational analyses to separately examine in HN-nap and NN-nap groups whether the performance was associated with the duration of each sleep stage as well as NREM spindle count and density in the nap preceding the task. We used a Fisher-*z*-transformation to test the significance of the difference between correlations. All statistical tests were two-tailed, significance level *p* < 0.05.

## Results

### Participants’ habitual sleep patterns

Actigraphically assessed sleep from the screening week and participant characteristics were analyzed for differences between the HN and NN groups (Table 1). Compared to the NN group, the HN group had significantly less nocturnal TIB on weekdays (mean ± SEM difference: 38.25 ± 9.16 min, *t*(89) = 4.177, *p* < 0.001), weekends (mean ± SEM difference: 30.56 ± 13.85 min, *t*(89) = 2.206, *p* = 0.030), and on average (mean ± SEM difference: 36.05 ± 7.78 min, *t*(89) = 4.631, *p* < 0.001), as well as less average nocturnal TST (mean ± SEM difference: 27.50 ± 7.76 min, *t*(88) = 3.544, *p* = 0.001).

**Table 1.** Characteristics of habitual and non-habitual nappers from screening week

	Habitual nappers		Non-habitual nappers		*t*/*χ*^2^	*p*
	Mean	*SD*	Mean	*SD*		
*n*	48	–	43	–	–	–
Age (years)	16.70	1.05	16.28	0.82	2.08	**0.040**
Sex (number of females)	26	–	22	–	–	
Caffeine (drinks per day)	0.52	0.68	0.84	1.04	1.76	0.081
Raven’s Progressive Matrices score	9.58	1.80	8.49	1.82	2.89	**0.005**
Epworth Sleepiness Scale score	8.21	3.57	7.81	3.12	0.56	0.578
PSQI global score	4.42	1.77	4.26	1.40	0.46	0.650
Morningness–Eveningness score	48.77	7.29	49.37	6.31	0.42	0.677
Beck Depression Inventory score	10.50	5.85	10.47	6.05	0.03	0.978
Beck Anxiety Inventory score	10.17	6.23	10.77	6.76	0.44	0.660
Actigraphy measures						
Nocturnal TIB on weekdays (h)	6.70	0.73	7.34	0.73	4.18	**<0.001**
Nocturnal TIB on weekends (h)	8.08	1.16	8.59	1.03	2.21	**0.030**
Nocturnal TIB on average (h)	7.09	0.61	7.69	0.62	4.63	**<0.001**
Nocturnal TST on weekdays (h)	5.37	0.65	5.70	1.16	1.74	0.085
Nocturnal TST on weekends (h)	6.51	1.16	6.93	0.94	1.84	0.070
Nocturnal TST on average (h)	5.69	0.58	6.15	0.65	3.54	**0.001**
Wake time on weekdays*	06:53	1.38	06:59	1.20	0.40	0.689
Wake time on weekends*	08:40	1.71	08:42	1.26	0.14	0.887
Wake time on average*	07:23	1.33	07:29	1.05	0.34	0.732
Bedtime on weekdays*	00:13	1.51	23:39	1.00	2.06	**0.042**
Bedtime on weekends*	00:35	1.14	00:08	0.95	2.01	**0.047**
Bedtime on average*	00:19	1.31	23:47	0.88	2.23	**0.028**

*Note*. *SD*, standard deviation; PSQI, the Pittsburgh Sleep Quality Index; TIB, time in bed; TST, total sleep time.

Bold values indicate *p* values < 0.05.

*Mean = 24 h clock time, *SD* = h.

Habitual nappers’ comparatively shorter nocturnal sleep duration appeared to be driven by their significantly later bedtimes on both weekdays (mean ± SEM difference: 33.71 ± 16.33 min, *t*(89) = 2.064, *p* = 0.042) and weekends (mean ± SEM difference: 26.78 ± 13.31 min, *t*(89) = 2.011, *p* = 0.047), as well as on average (mean ± SEM difference: 31.68 ± 14.22 min, *t*(89) = 2.228, *p* = 0.028) while wake times did not significantly differ between HN and NN groups (*p*s > 0.689).

Additionally, compared to the NN group, the HN group had significantly higher total scores on the Raven’s Progressive Matrices test (mean ± SEM difference: 1.10 ± 0.38, *t*(89) = 2.885, *p* = 0.005). There were no other significant differences from the screening variables between the two groups (*p*s > 0.070) except for age which showed a slight difference between the groups (mean ± SEM difference: 0.42 ± 0.20 years, *t*(89) = 2.08, *p* = 0.040).

## Nap Macroarchitecture

**Figure 2.**
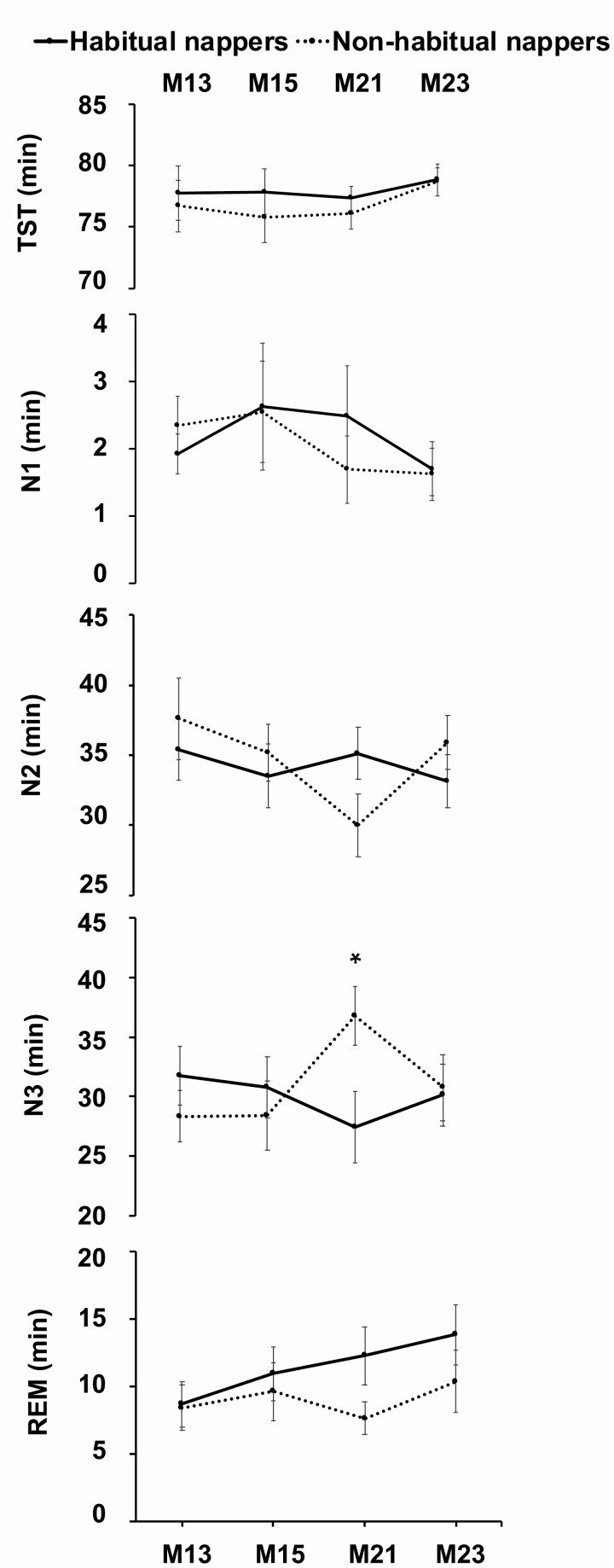
Nap sleep parameters assessed across polysomnography-monitored experimental naps: M1_3_ (third day of the first manipulation cycle, Four Mountains Task), M1_5_ (fifth day of the first manipulation cycle, picture encoding task), M2_1_ and M2_3_ (first and third days of the second manipulation cycle [no PSG performed on M2_2_], factual knowledge task). Means and standard errors are plotted separately for habitual nappers (solid line) and non-habitual nappers (dashed line) for total sleep time (TST) and duration of N1, N2, N3, and rapid-eye-movement (REM) sleep across each experimental nap period. *p < 0.05.

Overall, over the four experimental nap days that were assessed with polysomnography, habitual and non-habitual nappers did not generally differ in TST or the duration spent in N1, N2, N3, and REM, except for non-habitual nappers obtaining 9 min more N3 sleep during the nap preceding the first encoding session for the factual knowledge task (Figure 2, Supplementary Table 2). There were also no significant group differences in sleep latency, amount of WASO or SWA obtained. Furthermore, habitual and non-habitual nappers did not significantly differ in their likelihood of awakening from any particular sleep stage at the end of 90-min nap opportunity (*p* = 0.357).

## Four Mountains Task

**Figure 3.**
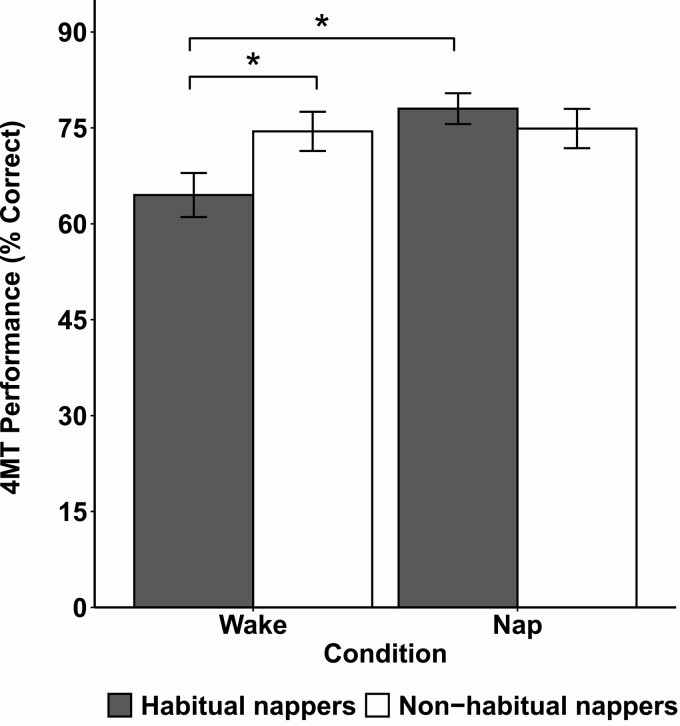
Four Mountains Task (4MT) performance for habitual (gray bars) and non-habitual nappers (white bars) in the experimental nap and wake conditions. Means and standard errors of the mean for the percentage of correct trials are plotted. *p < 0.05.

A two-way ANOVA showed a significant interaction between nap habit group (HN/NN) and condition (nap/wake) on 4MT task performance (*F*(1,87) = 4.649, *p* = 0.034) (Figure 3; Table 2). Habitual nappers who napped performed significantly better than habitual nappers who did not have a nap opportunity (HN-nap vs HN-wake: *t*(46) = 3.216, *p* = 0.002), while there was no significant difference between non-habitual nappers who napped and those who did not (NN-nap vs NN-wake: *t*(41) = 0.103, *p* = 0.918). Notably, amongst those who remained awake, habitual nappers performed significantly worse than non-habitual nappers (HN-wake vs NN-wake: *t*(44) = 2.146, *p* = 0.037). Further, these effects could not be accounted for by group differences in working memory and attention measured post-nap, as we found no significant nap habit (HN/NN) × condition (nap/wake) interactions for *n*-back performance and PVT lapses or median RT (Supplementary Analyses).

**Table 2.** Means (*SD*) and *F* values for the effects of habitual napping group (HN/NN) and condition (nap/wake) on task performance

	Habitual nappers		Non-habitual nappers		*F* values		
	Nap	Wake	Nap	Wake	Group	Condition	Group × condition
Four Mountains Task (%)	78.00 (11.81)	64.50 (16.83)	74.90 (14.13)	74.45 (14.39)	1.284	5.312*	4.649*
Picture encoding task (A′)	0.75 (0.07)	0.73 (0.07)	0.75 (0.07)	0.71 (0.06)	0.293	4.038*	0.216
Factual knowledge task (%)	89.92 (27.45)	65.08 (32.53)	71.24 (28.36)	50.05 (35.50)	6.657*	12.405*	0.078

* *p <* 0.05.

We next examined the relationship between task performance and duration spent in sleep stages during the nap preceding 4MT. Overall, there was a significant positive correlation between 4MT performance and amount of N3 (*r*_s_ = 0.373, *p* = 0.013, Supplementary Figure 1). In the non-habitual nappers, this positive correlation was significant (*r*_s_ = 0.457, *p* = 0.043), while the correlation in the habitual nappers although positive, was not significant (*r*_s_ = 0.256, *p* = 0.227). Group differences in correlations were not significant (*z* = −0.580, *p* = 0.562). There were no other sleep parameters (N1, N2, REM, SWA) that showed significant associations with 4MT for habitual and non-habitual nappers (*p*s > 0.072).

However, we found a significant negative association between overall spindle density and FMT performance (*r*_s_ = −0.434, *p* = 0.034) but not with spindle count (*r*_s_ = −0.339, *p* = 0.105). This association appeared to be driven by the habitual nappers (*r*_s_ = −0.434, *p* = 0.034) as it was not significant in the non-habitual nappers (*r*_s_ = −0.393, *p* = 0.096); however, the difference in correlations was not significant (*z* = −0.16, *p* = 0.873). In addition, N3 duration was significantly negatively correlated with spindle density in habitual nappers (*r*_s_ = −0.471, *p* = 0.02) but not in non-habitual nappers (*r*_s_ = −0.393, *p* = 0.096, *z* = −0.300, *p* = 0.764). The inverse relationship between spindles and N3 in habitual nappers may reflect the restorative nature of the nap [56] for habitual nappers who may be more reliant on a nap to restore performance.

### Picture encoding task

Performance accuracy for this task was assessed by participants’ picture recognition scores (A′) at retrieval. A two-way ANOVA with group (HN/NN) and condition (nap/wake) as between-subject factors revealed a significant main effect of condition (*F*(1,87) = 4.038, *p* = 0.048, Table 2), whereby those who napped after encoding had significantly higher A′ scores than those who remained awake. There was no significant main effect of group (*F*(1,87) = 0.293, *p* = 0.590) and no significant interaction (*F*(1,87) = 0.216, *p* = 0.643).

### Factual knowledge task

A two-way ANOVA with group (HN/NN) and condition (nap/wake) showed a significant main effect of condition (*F*(1,87) = 12.405, *p* = 0.001, Table 2) which indicated that participants who napped before learning had significantly better performance compared those who stayed awake. We also found a significant main effect of group (*F*(1,87) = 6.657, *p* = 0.012), whereby habitual nappers had significantly better performance than non-habitual nappers. This appeared to be driven by habitual nappers in the nap group having the highest memory scores. However, the group × condition interaction was not significant (*F*(1,87) = 0.078, *p* = 0.781).

## Discussion

The present study found that a 90-min mid-afternoon nap opportunity benefitted both habitual and non-habitual nappers’ long-term memory. This concurs with previous studies showing that a mid-afternoon nap can boost the learning of factual knowledge [7, 55], word pairs [57], and picture encoding [58] in adolescents. However, on a short-term hippocampal-dependent topographical memory task, naps appear to have an additional restorative function unique to habitual nappers. Further, our results show that the differential effects of nap habit extend to encoding, adding to the existing literature that hitherto had only assessed consolidation.

For a regular napper, a nap may be *necessary* to restore the deterioration in performance resulting from nap restriction. When these persons were denied a mid-afternoon nap, performance on a short-term topographical memory task was significantly degraded compared to non-habitual nappers who similarly remained awake. This is especially striking given that habitual nappers in our sample scored higher on non-verbal intelligence (Table 1) and as a group performed relatively better on the factual learning task compared to non-habitual nappers (Table 2). Despite this, they were more impaired by having to remain awake than their non-napping counterparts.

Specifically, adverse effects of nap restriction for habitual nappers were found only on a hippocampal-dependent topographical memory task designed to probe flexible allocentric spatial processing [51] (see Table 3 for comparisons between memory tasks). Interestingly, adverse effects for nap-restricted habitual nappers on spatial memory have also been observed in 3–6 year-olds. In that study, Kurdziel et al. [34] found that toddlers who napped more versus less frequently were also relatively disadvantaged on a short-term visuospatial task when deprived of a nap. Although all the memory tasks investigated here may be considered hippocampal-dependent to some extent, it is possible that topographical tasks requiring allocentric spatial processing may be especially dependent on the hippocampus [59]. Indeed, patients with hippocampal degeneration are impaired on the 4MT [51, 60–63], and hippocampal volume has been found to be positively correlated with performance in healthy adults [52]. Compared to non-habitual nappers, habitual nappers may be more reliant on naps for the restoration of hippocampal-dependent learning performance [21, 31]. Given that sleep has been suggested to promote the downscaling of hippocampal synaptic networks so as to free up capacity for later encoding [64, 65], staying awake may have had particularly adverse effects on habitual nappers’ ability to encode the position of objects relative to one another in space.

**Table 3.** Comparisons across the three memory tasks

Memory task	Memory type	Type of encoding	Encoding day	Encoding time	Encoding period	Retention interval	Retrieval day	Retrieval time (pm)
Four Mountains	ST topographical	Explicit	M1_3_	16:45	16 min	7 s	M1_3_	16:45
Picture encoding	LT episodic	Incidental	M1_5_	16:45	15 min	2 days	R1_2_	16:45
Factual knowledge	LT semantic	Explicit	M2_1_ – M2_3_	16:45	3 days	3 days	R2_1_	20:30

ST, short-term; LT, long-term; M1_3_, third day of the first manipulation cycle; M1_5_, fifth day of the first manipulation cycle; M2_1_–M2_3_, first to third day of the second manipulation cycle; R1_2_, second day of the first rest cycle; R2_1_, first day of the second rest cycle.

The 4MT was also a short-term memory task whereby retrieval followed encoding after a few seconds delay. In contrast, the other two memory tasks investigated long-term memory, testing both episodic and semantic memory separately. The factual knowledge task has particular ecological validity since it simulates the learning of structured knowledge over multiple sessions, akin to what a student would encounter in daily life. For these long-term memory tasks, whether one is a habitual napper does not appear to affect nap benefit. Although it remains uncertain in what way the short-term nature of the 4MT contributed to the differential effects observed, it is worth noting that compared to long-term memory tasks, the 4MT has been found to be particularly sensitive to sleep restriction [6, 66]. Future studies may investigate this further by comparing the effects of napping and staying awake in habitual and non-habitual nappers across short- and long-term memory tasks of the same memory domains (e.g. spatial memory, episodic memory).

Importantly, non-habitual nappers were *not disadvantaged* by taking a 90-min nap for any of the tasks. This is in contrast to a study that found that non-habitual nappers’ performance on a “cup and ball” motor memory task deteriorated after a short 20 min nap opportunity [32]. Methodological differences in the tasks used and age group studied could limit comparisons between studies. In particular, differences in the durations of the naps studied raise the question of whether nap macroarchitecture may have contributed to differences in outcomes. While our 90-min nap mainly comprised N2 and N3 sleep, participants who slept 20 min in Milner’s study [32] did not obtain any N3 or REM sleep. To investigate whether and how this contributed to the different outcomes of a nap for habitual and non-habitual nappers, future studies should seek to vary nap durations and consequently the duration of nap sleep stages obtained by habitual and non-habitual nappers.

It has been proposed that non-habitual nappers may eschew napping because of greater discomfort from sleep inertia upon waking [19, 28] and that this may be due to non-habitual nappers having more N3 sleep during a nap [29]. However, similar to McDevitt and colleagues [33], our habitual and non-habitual nappers did not significantly differ in post-nap subjective sleepiness and vigilance. This converges with our finding and that of others [32, 33] that habitual and non-nappers did not have significantly different amounts of N3 during the naps. We observed that non-habitual nappers obtained 9 mins more N3 sleep on one of our experimental days (*p* = 0.039), but this finding was not consistent across days. Further, non-nappers in our study did not have a higher likelihood of awakening from N3 at the end of the 90-min nap opportunity.

Although it could also be posited that non-habitual nappers do not nap because they are limited in their ability to fall asleep in the afternoon, the fact that all our participants managed to nap during the protocol suggests that nap habit in this age group may be influenced more by *opportunity* as opposed to *ability* to nap. It is worth noting that habitually and non-habitually napping young adults in McDevitt and colleagues’ 90 min nap protocol did not differ in sleep latency either, and both habitual and non-habitual nappers achieved an average of 80%–82% sleep efficiency [15]. Interestingly, Milner and colleagues reported that young adult non-habitual nappers in their study in fact had *shorter* sleep latency during a 20 min nap opportunity compared to habitual nappers [32]. Taken together, it appears that nap habit may not be a significant barrier to daytime napping, especially when nap opportunity is timed to coincide with the well-established propensity for sleepiness in the afternoon [67].

Previous literature has identified different reasons for why people nap [19, 68]. In our sample, although reasons for napping were not explicitly probed, sleep patterns measured during school term time suggest that naps were taken mainly for restorative reasons. Compared to non-habitual nappers, habitual nappers tended to have later bedtimes and shorter nocturnal TIB, indicating that they may have utilized naps to increase their total sleep opportunity across 24 h. Notably, later bedtimes did not appear to be driven by habitual nappers having a later chronotype (Table 1) and may instead be related to the pressure to delay bedtimes to complete homework [69]. For competitive societies where more than 60% of teens go to bed past midnight [20], naps may in fact be a common strategy (US: 40%–60% of teens nap on school days) to supplement short nocturnal sleep. Nonetheless, whether or not nocturnal sleep opportunity falls below or within recommendations, our work has shown that naps will benefit vigilance [39] and memory [6] across a range of students.

In summary, these lines of research converge to highlight the importance of implementing naps in an adolescent population. While delaying school start times has seen some success [70, 71], many societies remain deterred by the macrostructural adjustments required by disparate segments of the workforce. In addition, students’ persistent late bedtimes may limit the gains in TST that can be achieved with delayed rise times. As such, introducing a nap opportunity during the school day [7] may offer a complementary strategy to boost sleep health, learning, as well as protect those vulnerable to nap restriction.

## Limitations

The present study investigated a 90 min nap opportunity, which may arguably be too long to be practicable. However, this duration was used as sleep studies have traditionally utilized this nap length to allow for a full cycle of sleep and to examine any contributions of REM. Future studies should aim to test nap benefits in habitual and non-habitual nappers with shorter nap durations.

As we did not explicitly probe adolescents’ reasons for napping, we were unable to systematically examine the proportion of our sample who napped for restorative, appetitive, or medical reasons [19]. Pertinently, the reasons for why *non-nappers do not nap* remain unstudied. Although the present work was able to rule out inability to nap as a barrier to napping, future studies should include questionnaires to comprehensively assess reasons for both napping and not napping.

The present work did not objectively measure the duration of naps habitually taken by nappers. Hence, habitual nappers’ total sleep duration obtained over 24 h cannot be reliably estimated. To what extent habitual nappers are able to make up for their shorter nocturnal sleep with daytime naps on a regular basis remains uncertain. Future studies should seek to compare the total sleep duration obtained over 24 h between habitual and non-habitual nappers.

## Conclusions

Contrary to concerns that napping may be disadvantageous for non-habitual nappers [32], the present work found that an afternoon nap was beneficial for long-term memory even if one does not habitually nap. Importantly, naps were especially beneficial for habitual nappers performing a hippocampal-dependent short-term topographical memory task, as it restored the decline that would otherwise have been incurred without a nap. Our findings inform sleep strategies to boost and protect learning and memory in school-going adolescents and support the implementation of naps in educational settings.

## Supplementary Material

zsaa277_suppl_Supplementary_Materials

Suppl_Table_1

Suppl_Table_2

## Data Availability

The data underlying this article are available in the article and in its online supplementary material.
