# Erratum to: Memory performance following napping in habitual and non-habitual nappers

**DOI:** 10.1093/sleep/zsab038

**Published:** 2021-06-24

**Authors:** Ruth L F Leong, Nicole Yu, Ju Lynn Ong, Alyssa S C Ng, S Azrin Jamaluddin, James N Cousins, Nicholas I Y N Chee, Michael W L Chee

Upon the original publication of this article, errors in “Table 1. Characteristics of habitual and non-habitual nappers from screening week” were noted and list in this erratum.

Table 1 has been updated as follows online:

**Table:** Previous version Table 1. Characteristics of habitual and non-habitual nappers from screening week

	Habitual nappers		Non-habitual nappers		*t*/*χ*^2^	*p*
	Mean	*SD*	Mean	*SD*		
*n*	48	43	–	–	–	–
Age (years)	16.70	1.05	16.28	0.82	2.08	**0.040**
Gender (number of females)	26	22	–	0.28	0.777	
Caffeine (drinks per day)	0.52	0.68	0.84	1.04	1.76	0.081
Raven’s Progressive Matrices score	9.58	1.80	8.49	1.82	2.89	**0.005**
Epworth Sleepiness Scale score	8.21	3.57	7.81	3.12	0.56	0.578
PSQI global score	4.42	1.77	4.26	1.40	0.46	0.650
Morningness–Eveningness score	48.77	7.29	49.37	6.31	0.42	0.677
Beck Depression Inventory score	10.50	5.85	10.47	6.05	0.03	0.978
Beck Anxiety Inventory score	10.17	6.23	10.77	6.76	0.44	0.660
Actigraphy measures						
Nocturnal TIB on weekdays (h)	6.70	0.73	7.34	0.73	4.18	**<0.001**
Nocturnal TIB on weekends (h)	8.08	1.16	8.59	1.03	2.21	**0.030**
Nocturnal TIB on average (h)	7.09	0.61	7.69	0.62	4.63	**<0.001**
Nocturnal TST on weekdays (h)	5.37	0.65	5.70	1.16	1.74	0.085
Nocturnal TST on weekends (h)	6.51	1.16	6.93	0.94	1.84	0.070
Nocturnal TST on average (h)	5.69	0.58	6.15	0.65	3.54	**0.001**
Wake time on weekdays*	06:53	1.38	06:59	1.20	0.40	0.689
Wake time on weekends*	08:40	1.71	08:42	1.26	0.14	0.887
Wake time on average*	07:23	1.33	07:29	1.05	0.34	0.732
Bedtime on weekdays*	00:13	1.51	23:39	1.00	2.06	**0.042**
Bedtime on weekends*	00:35	1.14	00:08	0.95	2.01	**0.047**
Bedtime on average*	00:19	1.31	23:47	0.88	2.23	**0.028**

*Note*. *SD*, standard deviation; PSQI, the Pittsburgh Sleep Quality Index; TIB, time in bed; TST, total sleep time.

Bold values indicate *p* values < 0.05.

*Mean = 24 h clock time, *SD* = h.

**Table:** Corrected version Table 1. Characteristics of habitual and non-habitual nappers from screening week

	Habitual nappers		Non-habitual nappers		*t*/*χ*^2^	*p*
	Mean	*SD*	Mean	*SD*		
*n*	48	–	43	–	–	–
Age (years)	16.70	1.05	16.28	0.82	2.08	**0.040**
Sex (number of females)	26	–	22	–	–	–
Caffeine (drinks per day)	0.52	0.68	0.84	1.04	1.76	0.081
Raven’s Progressive Matrices score	9.58	1.80	8.49	1.82	2.89	**0.005**
Epworth Sleepiness Scale score	8.21	3.57	7.81	3.12	0.56	0.578
PSQI global score	4.42	1.77	4.26	1.40	0.46	0.650
Morningness–Eveningness score	48.77	7.29	49.37	6.31	0.42	0.677
Beck Depression Inventory score	10.50	5.85	10.47	6.05	0.03	0.978
Beck Anxiety Inventory score	10.17	6.23	10.77	6.76	0.44	0.660
Actigraphy measures						
Nocturnal TIB on weekdays (h)	6.70	0.73	7.34	0.73	4.18	**<0.001**
Nocturnal TIB on weekends (h)	8.08	1.16	8.59	1.03	2.21	**0.030**
Nocturnal TIB on average (h)	7.09	0.61	7.69	0.62	4.63	**<0.001**
Nocturnal TST on weekdays (h)	5.37	0.65	5.70	1.16	1.74	0.085
Nocturnal TST on weekends (h)	6.51	1.16	6.93	0.94	1.84	0.070
Nocturnal TST on average (h)	5.69	0.58	6.15	0.65	3.54	**0.001**
Wake time on weekdays*	06:53	1.38	06:59	1.20	0.40	0.689
Wake time on weekends*	08:40	1.71	08:42	1.26	0.14	0.887
Wake time on average*	07:23	1.33	07:29	1.05	0.34	0.732
Bedtime on weekdays*	00:13	1.51	23:39	1.00	2.06	**0.042**
Bedtime on weekends*	00:35	1.14	00:08	0.95	2.01	**0.047**
Bedtime on average*	00:19	1.31	23:47	0.88	2.23	**0.028**

*Note*. *SD*, standard deviation; PSQI, the Pittsburgh Sleep Quality Index; TIB, time in bed; TST, total sleep time.

Bold values indicate *p* values < 0.05.

*Mean = 24 h clock time, *SD* = h.

The publisher would like to apologize for any inconvenience caused to the reader.

