# Supplementary material for: Memory performance following napping in habitual and non-habitual nappers
Source: Sleep. 2020 Dec 12;44(6):zsaa277. doi: 10.1093/sleep/zsaa277 (PMC8193563; doi:10.1093/sleep/zsaa277)
Supplement: zsaa277_suppl_Supplementary_Materials [file zsaa277_suppl_supplementary_materials.docx]

**Memory Performance Following Napping In Habitual And Non-Habitual Nappers**

Ruth L. F. Leong^†a^, Nicole Yu^†a^, Ju Lynn Ong^a^, Alyssa S. C. Ng^a^, S. Azrin Jamaluddin^a^, James N. Cousins^b^, Nicholas I. Y. N. Chee^a^, Michael W. L. Chee*^a^

^a^Centre for Sleep and Cognition, Yong Loo Lin School of Medicine, National University of Singapore, Singapore

^b^Donders Institute for Brain, Cognition & Behaviour, Radboud University Medical Centre, 6525 EN, Nijmegen, The Netherlands

^†^Both first authors contributed equally to this work

*Corresponding author:

Dr. Michael W.L. Chee

Centre for Sleep and Cognition

NUS Yong Loo Lin School of Medicine,

MD1, 12 Science Drive 2

Singapore 117549

Phone: (+65) 66013199

E-mail: michael.chee@nus.edu.sg

**SUPPLEMENTARY ANALYSIS**

As part of an exploratory analysis, we examined whether the effect of a nap benefit on short-term topographical memory could be accounted for by working memory and attention. These were measured using the n-back task and psychomotor vigilance task (PVT), respectively.

**Methods**

*N-back*

Separate blocks of 1-back and 3-back tasks were performed three times a day as part of a test battery. A baseline period was used to determine that the groups were matched for working memory and executive function. In this task, a letter appeared centrally for 1000 ms, followed by a blank screen ITI of 3000 ms before the presentation of the next letter. For the 1-back task, participants were instructed to respond with a button press to indicate whether the current stimulus presented to them matched (Y) or did not match (N) the letter from the previous trial. For the 3-back task, they had to do the same, except to determine whether the current stimulus matched the letter which was presented three trials ago.

The match to mismatch ratio was 8:24. There were two performance indicators: A’ and B”. A’ indicated the ability to discriminate between matches and mismatches (range: 0-1; chance performance = 0.5). B” indicated the tendency towards liberal (B”D < 0) or conservative (B”D > 0) response bias (neutrality: B″ = 0). Afternoon A’ scores were used for analysis.

*PVT*

The PVT was a reaction time (RT) task used as an indication of sustained attention, and was performed in a 10 min continuous block. For the task, a counter appeared on the screen at random intervals between 2000 ms and 10000 ms. Participants had to respond to the counter by pressing on the keyboard space bar as quickly as possible. If no response was made within 10000 ms, an alerting beep played via headphones. Response speed (median RT) and lapses (responses > 500 ms) were measured, with afternoon performances being used for analysis.

**Results**

*N-back*

For the 1-back, a two-way ANOVA revealed no significant main effects of nap habituality group (HN/NN) and condition (Nap/Wake), or interaction on task performance (group: *F*(1,87) = 0.346, *p* = 0.558; condition: *F*(1,87) = 0.043, *p* = 0.837; group * condition: *F*(1,87) = 3.273, *p* = 0.074). Similarly, a two-way ANOVA performed on the 3-back showed no significant main effects or interaction (group: *F*(1,87) = 1.377, *p* = 0.244; condition: *F*(1,87) = 3.196, *p* = 0.077; group * condition: *F*(1,87) = 1.463, *p* = 0.230).

*PVT*

Two-way ANOVAs showed that there were no main effects of group (median RT: *F*(1,87) = 0.318, *p* = 0.574; lapses: *F*(1,87) = 0.992, *p* = 0.322) and no group * condition interactions (median RT: *F*(1,87) = 0.320, *p* = 0.573; lapses: *F*(1,87) = 0.645, *p* = 0.424). There were significant main effects of condition for median RT (*F*(1,87) = 365.403, *p <* 0.001) and lapses (*F*(1,87) = 10.580, *p* = 0.002), with those in the Nap condition having significantly slower median RTs (*t*(89) = 19.318, *p* < 0.001) and less lapses (*t*(89) = 3.231, *p* = 0.002) than the Wake condition.

**SUPPLEMENTARY TABLE 1.** Nap sleep architecture of Need for Sleep 4 (NFS4) and 5 (NFS5) participants in the Nap condition over the experimental days, measured by polysomnography.

NFS4 (*n* = 21) NFS5 (*n* = 24)

Duration (min) Mean SD Mean SD *t p*

M1_3_:

Total sleep time 80.41 2.90 74.41 13.14 2.04 0.047*

Stage 1 sleep 1.98 1.46 2.24 1.93 0.51 0.616

Stage 2 sleep 38.79 12.41 34.20 10.89 1.31 0.198

Stage 3 sleep 31.10 9.83 29.39 12.41 0.50 0.619

Rapid-eye movement sleep 8.55 8.06 8.59 7.58 0.17 0.987

Non-rapid eye movement sleep 71.86 8.69 65.83 11.50 1.95 0.058

Wake after sleep onset 1.38 1.56 4.65 10.90 1.36 0.181

Stage 2 sleep latency 8.76 3.01 11.53 5.39 1.98 0.058

M1_5_:

Total sleep time 78.95 6.88 74.94 10.38 1.50 0.142

Stage 1 sleep 3.71 4.93 1.57 2.69 1.82 0.076

Stage 2 sleep 33.33 10.50 35.20 9.95 0.60 0.549

Stage 3 sleep 28.24 13.10 30.94 12.49 0.70 0.488

Rapid-eye movement sleep 13.67 10.63 7.24 7.90 2.29 0.027*

Non-rapid eye movement sleep 65.29 11.93 67.70 9.51 0.74 0.461

Wake after sleep onset 2.36 3.51 2.44 6.58 0.05 0.962

Stage 2 sleep latency 9.19 5.94 12.50 8.18 1.52 0.135

M2_1_:

Total sleep time 77.83 5.15 75.83 5.49 1.25 0.217

Stage 1 sleep 2.91 4.01 1.42 1.91 1.62 0.112

Stage 2 sleep 33.50 10.16 32.06 9.70 0.49 0.630

Stage 3 sleep 32.41 15.63 31.27 12.48 0.27 0.788

Rapid-eye movement sleep 9.02 10.13 11.08 8.13 0.76 0.454

Non-rapid eye movement sleep 68.81 10.70 64.75 9.21 1.35 0.183

Wake after sleep onset 2.64 4.72 1.27 1.52 1.35 0.185

Stage 2 sleep latency 10.00 3.59 13.15 6.27 2.02 0.049*

M2_3_:

Total sleep time 80.29 5.20 77.35 6.00 1.73 0.091

Stage 1 sleep 1.91 1.87 1.44 1.80 0.85 0.401

Stage 2 sleep 34.83 9.59 34.13 8.71 0.26 0.800

Stage 3 sleep 31.21 11.77 29.72 13.19 0.40 0.694

Rapid-eye movement sleep 12.33 9.18 12.07 12.05 0.08 0.935

Non-rapid eye movement sleep 67.95 10.12 65.28 12.82 0.76 0.450

Wake after sleep onset 1.81 2.09 0.83 0.83 2.02 0.054

Stage 2 sleep latency 8.33 5.15 11.67 5.44 2.09 0.043*

Note. SD = standard deviation. M1_3_ = Third day of the first manipulation cycle; M1_5_: Fifth day of the first manipulation cycle; M2_1_: First day of the second manipulation cycle; M2_3_: Third day of the second manipulation cycle. *Marginally significant due to one participant napping for 32 mins out of the 90 min nap opportunity.

**SUPPLEMENTARY TABLE 2.** Nap macroarchitecture of habitual and non-habitual nappers in the Nap condition over the experimental days, measured by polysomnography.

Habitual nappers (*n* = 24) Non-habitual nappers (*n* = 21)

Duration (min) Mean SD Mean SD *t p*

M1_3_:

Total sleep time 77.75 10.86 76.70 9.28 0.35 0.731

Stage 1 sleep 1.92 1.46 2.35 1.97 0.84 0.408

Stage 2 sleep 35.38 10.64 37.60 13.10 0.62 0.537

Stage 3 sleep 31.77 12.20 28.33 9.74 1.02 0.313

Rapid-eye movement sleep 8.69 8.15 8.43 7.38 0.11 0.912

Non-rapid eye movement sleep 69.06 11.78 68.28 9.24 0.47 0.809

Wake after sleep onset 3.13 8.71 3.05 7.38 0.03 0.976

Stage 2 sleep latency 9.54 4.26 10.79 4.77 0.87 0.389

M1_5_:

Total sleep time 77.85 8.84 75.76 9.29 0.76 0.450

Stage 1 sleep 2.63 4.57 2.55 3.43 0.07 0.947

Stage 2 sleep 33.52 10.88 35.17 9.45 0.53 0.597

Stage 3 sleep 30.76 12.34 28.43 13.29 0.60 0.549

Rapid-eye movement sleep 10.94 9.74 9.62 9.95 0.44 0.660

Non-rapid eye movement sleep 66.91 10.93 66.14 10.64 0.24 0.814

Wake after sleep onset 1.72 3.04 3.14 6.98 0.89 0.377

Stage 2 sleep latency 10.61 8.49 11.26 5.94 0.29 0.771

M2_1_:

Total sleep time 77.33 4.86 76.12 5.95 0.74 0.462

Stage 1 sleep 2.48 3.72 1.69 2.30 0.84 0.405

Stage 2 sleep 35.13 9.03 30.00 10.20 1.79 0.081

Stage 3 sleep 27.44 14.57 36.79 11.43 2.37 **0.022**

Rapid-eye movement sleep 12.29 11.00 7.64 5.48 1.76 0.086

Non-rapid eye movement sleep 65.04 11.34 68.48 8.16 1.15 0.256

Wake after sleep onset 2.38 4.45 1.38 1.63 0.97 0.339

Stage 2 sleep latency 10.75 4.01 12.74 6.56 1.24 0.220

M2_3_:

Total sleep time 78.83 6.33 78.67 5.23 0.09 0.928

Stage 1 sleep 1.70 1.92 1.62 1.77 0.14 0.892

Stage 2 sleep 33.15 9.17 35.91 8.88 1.01 0.319

Stage 3 sleep 30.13 12.47 30.76 12.64 0.17 0.868

Rapid-eye movement sleep 13.85 10.53 10.38 10.74 1.08 0.286

Non-rapid eye movement sleep 64.98 10.70 68.29 12.45 0.95 0.349

Wake after sleep onset 1.20 1.66 1.41 1.61 0.42 0.674

Stage 2 sleep latency 10.41 6.27 9.71 4.66 0.42 0.679

Note. SD = standard deviation. M1_3_ = Third day of the first manipulation cycle; M1_5_: Fifth day of the first manipulation cycle; M2_1_: First day of the second manipulation cycle; M2_3_: Third day of the second manipulation cycle.

**SUPPLEMENTARY FIGURE 1.** Associations between Four Mountains Task (4MT) performance and amount of N3 obtained during the preceding nap are plotted separately for habitual nappers (black circles) and non-habitual nappers (grey triangles). *p < 0.05.
