# Supplementary material for: Memory performance following napping in habitual and non-habitual nappers
Source: Sleep. 2020 Dec 12;44(6):zsaa277. doi: 10.1093/sleep/zsaa277 (PMC8193563; doi:10.1093/sleep/zsaa277)
Supplement: Suppl_Table_1 [file suppl_table_1.docx]

**Memory Performance Following Napping In Habitual And Non-Habitual Nappers**

Ruth L. F. Leong^†a^, Nicole Yu^†a^, Ju Lynn Ong^a^, Alyssa S. C. Ng^a^, S. Azrin Jamaluddin^a^, James N. Cousins^b^, Nicholas I. Y. N. Chee^a^, Michael W. L. Chee*^a^

^a^Centre for Sleep and Cognition, Yong Loo Lin School of Medicine, National University of Singapore, Singapore

^b^Donders Institute for Brain, Cognition & Behaviour, Radboud University Medical Centre, 6525 EN, Nijmegen, The Netherlands

^†^Both first authors contributed equally to this work

*Corresponding author:

Dr. Michael W.L. Chee

Centre for Sleep and Cognition

NUS Yong Loo Lin School of Medicine,

MD1, 12 Science Drive 2

Singapore 117549

Phone: (+65) 66013199

E-mail: michael.chee@nus.edu.sg

**SUPPLEMENTARY TABLE 1.** Nap sleep architecture of Need for Sleep 4 (NFS4) and 5 (NFS5) participants in the Nap condition over the experimental days, measured by polysomnography.

NFS4 (*n* = 21) NFS5 (*n* = 24)

Duration (min) Mean SD Mean SD *t p*

M1_3_:

Total sleep time 80.41 2.90 74.41 13.14 2.04 0.047*

Stage 1 sleep 1.98 1.46 2.24 1.93 0.51 0.616

Stage 2 sleep 38.79 12.41 34.20 10.89 1.31 0.198

Stage 3 sleep 31.10 9.83 29.39 12.41 0.50 0.619

Rapid-eye movement sleep 8.55 8.06 8.59 7.58 0.17 0.987

Non-rapid eye movement sleep 71.86 8.69 65.83 11.50 1.95 0.058

Wake after sleep onset 1.38 1.56 4.65 10.90 1.36 0.181

Stage 2 sleep latency 8.76 3.01 11.53 5.39 1.98 0.058

M1_5_:

Total sleep time 78.95 6.88 74.94 10.38 1.50 0.142

Stage 1 sleep 3.71 4.93 1.57 2.69 1.82 0.076

Stage 2 sleep 33.33 10.50 35.20 9.95 0.60 0.549

Stage 3 sleep 28.24 13.10 30.94 12.49 0.70 0.488

Rapid-eye movement sleep 13.67 10.63 7.24 7.90 2.29 0.027*

Non-rapid eye movement sleep 65.29 11.93 67.70 9.51 0.74 0.461

Wake after sleep onset 2.36 3.51 2.44 6.58 0.05 0.962

Stage 2 sleep latency 9.19 5.94 12.50 8.18 1.52 0.135

M2_1_:

Total sleep time 77.83 5.15 75.83 5.49 1.25 0.217

Stage 1 sleep 2.91 4.01 1.42 1.91 1.62 0.112

Stage 2 sleep 33.50 10.16 32.06 9.70 0.49 0.630

Stage 3 sleep 32.41 15.63 31.27 12.48 0.27 0.788

Rapid-eye movement sleep 9.02 10.13 11.08 8.13 0.76 0.454

Non-rapid eye movement sleep 68.81 10.70 64.75 9.21 1.35 0.183

Wake after sleep onset 2.64 4.72 1.27 1.52 1.35 0.185

Stage 2 sleep latency 10.00 3.59 13.15 6.27 2.02 0.049*

M2_3_:

Total sleep time 80.29 5.20 77.35 6.00 1.73 0.091

Stage 1 sleep 1.91 1.87 1.44 1.80 0.85 0.401

Stage 2 sleep 34.83 9.59 34.13 8.71 0.26 0.800

Stage 3 sleep 31.21 11.77 29.72 13.19 0.40 0.694

Rapid-eye movement sleep 12.33 9.18 12.07 12.05 0.08 0.935

Non-rapid eye movement sleep 67.95 10.12 65.28 12.82 0.76 0.450

Wake after sleep onset 1.81 2.09 0.83 0.83 2.02 0.054

Stage 2 sleep latency 8.33 5.15 11.67 5.44 2.09 0.043*

Note. SD = standard deviation. M1_3_ = Third day of the first manipulation cycle; M1_5_: Fifth day of the first manipulation cycle; M2_1_: First day of the second manipulation cycle; M2_3_: Third day of the second manipulation cycle. *Marginally significant due to one participant napping for 32 mins out of the 90 min nap opportunity.
