# Supplementary material for: Memory performance following napping in habitual and non-habitual nappers
Source: Sleep. 2020 Dec 12;44(6):zsaa277. doi: 10.1093/sleep/zsaa277 (PMC8193563; doi:10.1093/sleep/zsaa277)
Supplement: Suppl_Table_2 [file suppl_table_2.docx]

**Memory Performance Following Napping In Habitual And Non-Habitual Nappers**

Ruth L. F. Leong^†a^, Nicole Yu^†a^, Ju Lynn Ong^a^, Alyssa S. C. Ng^a^, S. Azrin Jamaluddin^a^, James N. Cousins^b^, Nicholas I. Y. N. Chee^a^, Michael W. L. Chee*^a^

^a^Centre for Sleep and Cognition, Yong Loo Lin School of Medicine, National University of Singapore, Singapore

^b^Donders Institute for Brain, Cognition & Behaviour, Radboud University Medical Centre, 6525 EN, Nijmegen, The Netherlands

^†^Both first authors contributed equally to this work

*Corresponding author:

Dr. Michael W.L. Chee

Centre for Sleep and Cognition

NUS Yong Loo Lin School of Medicine,

MD1, 12 Science Drive 2

Singapore 117549

Phone: (+65) 66013199

E-mail: michael.chee@nus.edu.sg

**SUPPLEMENTARY TABLE 2.** Nap macroarchitecture of habitual and non-habitual nappers in the Nap condition over the experimental days, measured by polysomnography.

Habitual nappers (*n* = 24) Non-habitual nappers (*n* = 21)

Duration (min) Mean SD Mean SD *t p*

M1_3_:

Total sleep time 77.75 10.86 76.70 9.28 0.35 0.731

Stage 1 sleep 1.92 1.46 2.35 1.97 0.84 0.408

Stage 2 sleep 35.38 10.64 37.60 13.10 0.62 0.537

Stage 3 sleep 31.77 12.20 28.33 9.74 1.02 0.313

Rapid-eye movement sleep 8.69 8.15 8.43 7.38 0.11 0.912

Non-rapid eye movement sleep 69.06 11.78 68.28 9.24 0.47 0.809

Wake after sleep onset 3.13 8.71 3.05 7.38 0.03 0.976

Stage 2 sleep latency 9.54 4.26 10.79 4.77 0.87 0.389

M1_5_:

Total sleep time 77.85 8.84 75.76 9.29 0.76 0.450

Stage 1 sleep 2.63 4.57 2.55 3.43 0.07 0.947

Stage 2 sleep 33.52 10.88 35.17 9.45 0.53 0.597

Stage 3 sleep 30.76 12.34 28.43 13.29 0.60 0.549

Rapid-eye movement sleep 10.94 9.74 9.62 9.95 0.44 0.660

Non-rapid eye movement sleep 66.91 10.93 66.14 10.64 0.24 0.814

Wake after sleep onset 1.72 3.04 3.14 6.98 0.89 0.377

Stage 2 sleep latency 10.61 8.49 11.26 5.94 0.29 0.771

M2_1_:

Total sleep time 77.33 4.86 76.12 5.95 0.74 0.462

Stage 1 sleep 2.48 3.72 1.69 2.30 0.84 0.405

Stage 2 sleep 35.13 9.03 30.00 10.20 1.79 0.081

Stage 3 sleep 27.44 14.57 36.79 11.43 2.37 **0.022**

Rapid-eye movement sleep 12.29 11.00 7.64 5.48 1.76 0.086

Non-rapid eye movement sleep 65.04 11.34 68.48 8.16 1.15 0.256

Wake after sleep onset 2.38 4.45 1.38 1.63 0.97 0.339

Stage 2 sleep latency 10.75 4.01 12.74 6.56 1.24 0.220

M2_3_:

Total sleep time 78.83 6.33 78.67 5.23 0.09 0.928

Stage 1 sleep 1.70 1.92 1.62 1.77 0.14 0.892

Stage 2 sleep 33.15 9.17 35.91 8.88 1.01 0.319

Stage 3 sleep 30.13 12.47 30.76 12.64 0.17 0.868

Rapid-eye movement sleep 13.85 10.53 10.38 10.74 1.08 0.286

Non-rapid eye movement sleep 64.98 10.70 68.29 12.45 0.95 0.349

Wake after sleep onset 1.20 1.66 1.41 1.61 0.42 0.674

Stage 2 sleep latency 10.41 6.27 9.71 4.66 0.42 0.679

Note. SD = standard deviation. M1_3_ = Third day of the first manipulation cycle; M1_5_: Fifth day of the first manipulation cycle; M2_1_: First day of the second manipulation cycle; M2_3_: Third day of the second manipulation cycle.
